# *Mycobacterium tuberculosis* and Rifampin Resistance, United Kingdom

**DOI:** 10.3201/eid1205.041339

**Published:** 2006-05

**Authors:** I-Ching Sam, Francis Drobniewski, Philip More, Melanie Kemp, Timothy Brown

**Affiliations:** * Health Protection Agency, London, United Kingdom

**Keywords:** Tuberculosis, multidrug resistant, rifampin, DNA probes, United Kingdom, research

## Abstract

The United Kingdom Health Protection Agency Mycobacterium Reference Unit offers a national "Fastrack" molecular service for detecting *Mycobacterium tuberculosis* complex (MTBC) and rifampin resistance by using the INNO-LiPA Rif.TB assay. We analyzed the service in a routine, nontrial context of 1,997 primary clinical specimens, including 658 nonrespiratory specimens. The overall adjusted concordance, sensitivity, specificity, positive predictive value, and negative predictive value for detecting MTBC were 91.2%, 85.2%, 96.2%, 95.7%, and 86.7%, respectively (unadjusted, 86.7%, 85.2%, 88.2%, 86.9%, and 86.7%), when false-positive samples from patients (n = 83) with a known microbiologic diagnosis of MTBC or patients receiving current or recent antituberculous treatment were excluded. The parameters for detecting rifampin resistance were 99.1%, 95.0%, 99.6%, 92.7%, and 99.7%, respectively. The assay enabled earlier diagnosis of MTBC and rifampin resistance (15.2 days) compared with culture-based techniques (30.7 days).

## Abstract 2

A national diagnostic service identified *M*. *tuberculosis* and rifampin resistance in primary clinical specimens faster than conventional techniques.

The increasing incidence of multidrug-resistant tuberculosis (MDRTB), defined as resistance to at least rifampin and isoniazid, is a notable global health problem (*1*). The rapid identification of patients with MDRTB enables early institution of appropriate treatment, which is associated with improved survival (*2**,**3*), and infection control procedures to minimize risk of transmission (*4*). The Centers for Disease Control and Prevention recommends that the culture/identification and susceptibility testing of *Mycobacterium tuberculosis* complex (MTBC) be completed within 21 and 30 days of specimen receipt, respectively (*5*). Molecular assays based on the genetics of drug resistance may considerably reduce these turnaround times.

In the United Kingdom, 82.5% of rifampin-resistant isolates are also resistant to isoniazid (*6*), making rifampin resistance a useful surrogate marker for MDRTB. Most rifampin-resistant MTBC strains have mutations in an 81-bp region of the *rpoB* gene that encodes the RNA polymerase β subunit (*7*). This region is therefore an ideal target for molecular tests for rifampin resistance.

The United Kingdom Health Protection Agency Mycobacterium Reference Unit (MRU) offers a national molecular diagnostic service (Fastrack) for detection of MTBC and rifampin resistance (*8*) by using the INNO-LiPA Rif.TB assay (Innogenetics, Zwijndrecht, Belgium) and supplemented by DNA sequencing as needed. This assay is based on reverse hybridization between *rpoB* amplicons and membrane-bound capture probes (1 specific for MTBC, 5 overlapping wild-type probes spanning the *rpoB* target region, and 4 of the most common mutations). Genotypic resistance is indicated by absence of hybridization with wild-type probes or hybridization with resistance mutation probes (*9*).

A review of the line probe assay (LiPA) (*10*) found that most previous evaluations focused on mycobacterial isolates and culture-positive (mainly respiratory) specimens (*9**,**11**–**14*), but relatively little data exist on nonrespiratory and smear-negative specimens, which are often collected in routine clinical practice (*8**,**15**,**16*). The Fastrack service was initially targeted at smear-positive respiratory samples and mycobacterial isolates, but in response to widespread demand from other laboratories, was extended to all specimens, regardless of acid-fast bacilli (AFB) status. In January 2002, an in-house polymerase chain reaction (PCR) assay targeting the IS*6110* insertion sequence (*17*) replaced LiPA for testing cerebrospinal fluid (CSF) samples. Therefore, CSF samples were not included in this study. This study evaluated LiPA in the context of a nontrial clinical service in one of the largest reported samples of 1,997 primary clinical specimens (including 658 nonrespiratory) and 290 clinical isolates tested from 1999 to 2002.

## Materials and Methods

### Clinical Specimens

From January 1999 through December 2002, the MRU received 2,287 consecutive non-CSF specimens from 2,110 patients (comprising 1,997 primary clinical specimens and 290 clinical isolates) from 152 centers in the United Kingdom and Ireland for Fastrack analysis. Specimens are submitted for analysis at the discretion of individual referring laboratories, usually when the diagnosis of MTBC is uncertain or when rifampin resistance is suspected. When multiple specimens were received from a single patient, each specimen was processed separately. Of the primary specimens, 1,339 respiratory specimens were sputum, bronchial washings, and bronchial and tracheal aspirates; 658 were nonrespiratory specimens. Samples were received only on weekdays, and routine processing and culture were initiated within 24 hours of receipt. Turnaround times for completion of analysis, culture, and identification of MTBC and drug-susceptibility testing were calculated from date of specimen receipt (*5*).

### Routine Microscopy, Culture, Identification, and Susceptibility Testing

Samples were decontaminated by using the NaOH/N-acetyl-L-cysteine method in a 2-mL suspension, and AFB staining was performed with auramine-phenol and the Ziehl-Neelsen procedure (*18**,**19*). DNA was extracted from 1 mL of decontaminated specimen by using a previously described chloroform extraction technique (*20*), and the remaining 1 mL was added to 1 MB/BacT rapid culture vial (bioMérieux UK Ltd., Basingstoke, UK) and 1 Lowenstein-Jensen slope. Cultures were incubated for at least 8 weeks. Mycobacterial cultures were identified by microscopic and macroscopic appearances, biochemical tests, and DNA hybridization with Accuprobe (GenProbe, San Diego, CA, USA). Drug-susceptibility testing was carried out by the resistance ratio method (*18*).

### LiPA

LiPA was performed according to manufacturer's instructions. The first round of a nested PCR was performed with 10 μL of DNA extract and outer primers (LiPA OP1, 5´-GAGAATTCGGTCGGCGAGCTGATCC-3´ and LiPA OP2, 5´-CGAAGCTTGACCCGCGCGTACACC-3´) for 30 cycles at 95°C for 60 s, 58°C for 30 s, and 72°C for 90 s. One microliter of first-round product was transferred to a 40-μL PCR mixture containing inner primers (LiPA IP1, 5´-GGTCGGCATGTCGCGGATGG-3´ and LiPA IP2, 5´-GCACGTCGCGGACCTCCAGC-3´), which were biotinylated at the 5´ end, for the second round of amplification for 30 cycles at 95°C for 20 s, 65°C for 30 s, and 72°C for 30 s. Each PCR run included a duplicate and an inhibition control (100 genome copies of *Mycobacterium bovis* bacillus Calmette-Guérin [BCG]) for each sample, 5 extracted, water, negative controls, decontaminated, extracted, negative and positive controls (a known culture-positive clinical sample), and a positive control with a low amount of DNA (10 genome copies of BCG in 10 μL). A 260-bp band on agarose gel electrophoresis confirmed successful amplification. The hybridization assay to determine genotypic rifampin resistance was then performed and analyzed as previously described (*13*). The MTBC result was then reported as positive (accompanied by a rifampin-susceptibility result), negative, equivocal, or inhibited. Results were considered equivocal if a sample tested PCR positive on 1 of 2 duplicates on 2 separate occasions. Extracted DNA was stored for retesting equivocal and inhibited results and for future resolution of discrepant susceptibility results.

### Sequencing of *rpoB* PCR Product

Cultures of MTBC with discordant rifampin-susceptibility results by phenotypic and LiPA testing underwent automated sequencing of the *rpoB* PCR products with either the Long Read Tower System (Visible Genetics, Suwanee, GA, USA) or the CEQ 8000 Genetic Analysis System (Beckman Coulter, High Wycombe, UK). DNA was extracted from cultures and amplified in a PCR containing the outer primers OP1 and OP2 and sequenced with the inner primers IP1 and IP2.

### Statistical Analysis

Data were entered into Microsoft Access (Microsoft Corp., Redmond, WA, USA) and analyzed with Microsoft Excel. Detection of MTBC and rifampin resistance by LiPA was compared with results by the accepted standards of culture and phenotypic susceptibility testing. Concordance, sensitivity, specificity, positive predictive value (PPV), and negative predictive value (NPV) were calculated. We excluded 85 (3.7%) samples from primary analysis because LiPA results could not be compared with culture results. These samples had equivocal PCR results (n = 27, 1.2%), were inhibitory to PCR (n = 22, 1.0%), could not be cultured (e.g., because of insufficient volume or histologic samples embedded in paraffin wax; n = 6, 0.3%), or were contaminated with bacteria or fungi (n = 30, 1.3%).

## Results

### Microscopy and Culture

Of the primary specimens tested by LiPA, the AFB smear microscopy was positive in 1,137 (56.9%), negative in 821 (41.1%), and not performed in 39 (2.0%). Specimen types are shown in Tables 1 and 2. Culture identification and drug susceptibility results are shown in Table 3. MTBC was cultured from 941 (47.1%) of 1,997 primary samples and 238 (82.1%) of 290 isolates. In 3 cases, both MTBC and nontuberculous mycobacteria were cultured. A total of 1,178 *M*. *tuberculosis*, 10 *M*. *bovis* (including 1 BCG), and 1 *M*. *africanum* cultures were identified in the 4-year study period. During this time, 6,500–7,000 cases of tuberculosis were reported in the United Kingdom per year, including 4,500–5,000 reported to be culture positive (*6*). The times taken to culture MTBC from primary specimens are shown in Table 4. There were 223 nontuberculous mycobacteria isolates: 80 *M*. *avium* complex, 38 *M*. *kansasii*, 26 *M*. *xenopi*, 23 *M*. *malmoense*, 20 *M*. *chelonae*, 10 *M*. *fortuitum*, 3 *M*. *abscessus*, 3 *M*. *marinum*, 2 *M*. *gordonae*, 2 *M*. *simiae*, 2 *M*. *terrae*, 1 *M*. *szulgai*, 1 *M*. *vaccae*, and 12 unidentified *Mycobacterium* species.

**Table 1:** Results of LiPA compared with culture in detecting MTBC in primary clinical specimens*

Sample and AFB smear result	No. positive/no. tested (%)					
	Concordance	Sensitivity	Specificity	PPV	NPV	Mean days saved
All primary LiPA	1,667/1,922 (86.7)	782/918 (85.2)	886/1,004 (88.2)	782/900 (86.9)	886/1,022 (86.7)	15.2
Positive	960/1,099 (87.4)	747/798 (93.6)	213/301 (70.8)	747/835 (89.5)	213/264 (80.7)	14.8
Negative	679/792 (85.7)	35/119 (29.4)	644/673 (95.7)	35/64 (54.7)	644/728 (88.5)	22.1
Not done	28/31 (90.3)	0/1 (0)	29/30 (96.7)	0/1 (0)	29/30 (96.7)	–
All primary LiPA (adjusted values)†	1,667/1,828 (91.2)	782/918 (85.2)	886/921 (96.2)	782/817 (95.7)	886/1,022 (86.7)	15.2
Positive	960/1,028 (93.4)	747/798 (93.6)	213/232 (91.8)	747/766 (97.5)	213/264 (80.7)	14.8
Negative	679/771 (88.1)	35/119 (29.4)	644/659 (97.7)	35/50 (70.0)	644/728 (88.5)	22.1
Not done	28/29 (96.6)	0/1 (0)	29/30 (96.7)	0/1 (0)	29/30 (96.7)	–
Respiratory	1,168/1,298 (90.0)	672/738 (91.1)	496/560 (88.6)	672/736 (91.3)	496/562 (88.3)	14.7
Positive	827/915 (90.4)	657/696 (94.4)	170/219 (77.6)	657/706 (93.1)	170/209 (81.3)	14.5
Negative	328/369 (88.9)	15/42 (35.7)	313/327 (95.7)	15/29 (51.7)	313/340 (92.1)	20.5
Not done	13/14 (92.9)	–	13/14 (92.9)	0/1 (0.0)	13/13 (100.0)	–
Nonrespiratory	499/624 (80.0)	110/180 (61.1)	390/444 (87.8)	110/164 (67.1)	390/960 (84.8)	18.3
Positive	133/184 (72.3)	90/102 (88.2)	43/82 (52.4)	90/129 (69.8)	43/55 (78.2)	17.2
Negative	351/423 (83.0)	20/77 (26.0)	331/346 (95.7)	20/35 (57.1)	331/388 (85.3)	23.4
Not done	15/17 (88.2)	0/1 (0)	16/16 (100.0)	–	16/17 (94.1)	–-
Biopsy specimen‡	92/108 (85.2)	13/26 (50.0)	79/82 (96.3)	13/16 (81.3)	79/92 (85.9)	22.4
Positive	19/21 (90.5)	10/10 (100.0)	9/11 (81.8)	10/12 (83.3)	9/9 (100.0)	24.1
Negative	72/86 (83.7)	3/16 (18.8)	69/70 (98.6)	3/4 (75.0)	69/82 (84.1)	16.7
Not done	1/1 (100.0)	–	1/4 (25.0)	–	1/1 (100.0)	–
Gastric aspirate	17/18 (94.4)	4/5 (80.0)	13/13 (100.0)	4/4 (100.0)	13/14 (92.9)	16.8
Positive	8/8 (100.0)	4/4 (100.0)	4/4 (100.0)	4/4 (100.0)	4/4 (100.0)	16.8
Negative	9/10 (90.0)	0/1 (0.0)	9/9 (100.0)	–	9/10 (90.0)	–
Not done	–	–	–	–	–	–
Lymph node	103/142 (72.5)	50/68 (73.5)	53/74 (71.6)	50/71 (70.4)	53/71 (74.6)	18.7
Positive	54/79 (68.4)	42/48 (87.5)	12/31 (38.7)	42/61 (68.9)	12/18 (66.7)	16.6
Negative	48/62 (77.4)	8/20 (40.0)	40/42 (95.2)	8/10 (80.0)	40/52 (76.9)	29.6
Not done	1/1 (100.0)	–	1/1 (100.0)	–	1/1 (100.0)	–
Pleural fluid	84/107 (78.5)	5/23 (21.7)	79/84 (94.0)	5/10 (50.0)	79/97 (81.4)	26.8
Positive	6/10 (60.0)	4/5 (80.0)	2/5 (40.0)	4/7 (57.1)	2/3 (66.7)	23.5
Negative	77/97 (79.4)	1/18 (5.6)	76/78 (97.4)	1/3 (33.3)	76/93 (81.7)	40.0
Not done	1/1 (100.0)	–	1/1 (100.0)	–	1/1 (100.0)	–
Psoas abscess	8/15 (53.3)	3/6 (50.0)	5/9 (55.6)	3/7 (42.9)	5/8 (62.5)	19.0
Positive	2/3 (66.7)	2/2 (100.0)	0/1 (0.0)	2/3 (66.7)	–	17.0
Negative	6/12 (50.0)	1/4 (25.0)	5/8 (62.5)	1/4 (25.0)	5/8 (62.5)	23.0
Not done	–	–	–	–	–	–
Vertebral aspirate	26/30 (86.7)	10/12 (83.3)	16/18 (88.9)	10/12 (83.3)	16/18 (88.9)	15.4
Positive	9/10 (90.0)	8/8 (100.0)	1/2 (50.0)	8/9 (88.9)	1/1 (100.0)	14.5
Negative	17/19 (89.5)	2/3 (66.7)	15/16 (93.8)	2/3 (66.7)	15/16 (93.8)	19.0
Not done	0/1 (0.0)	0/1 (0.0)	–	–	0/1 (0.0)	–
Other§	169/204 (82.8)	25/40 (62.5)	145/164 (88.4)	25/44 (56.8)	145/160 (90.6)	15.2
Positive	35/53 (66.0)	20/25 (80.0)	15/28 (53.6)	20/33 (60.6)	15/20 (75.0)	15.1
Negative	122/138 (88.4)	5/15 (33.3)	117/123 (95.1)	5/11 (45.5)	117/127 (92.1)	15.8
Not done	12/13 (92.3)	–	13/13 (100.0)	–	13/13 (100.0)	–

*MTBC excludes 75 specimens containing substances inhibitory to the polymerase chain reaction (PCR), PCR-equivocal results, and samples with no definitive culture results (i.e., contaminated or not done). LiPA, line probe assay; MTBC, *Mycobacterium tuberculosis* complex; AFB, acid-fast bacilli; PPV, positive predictive value; NPV, negative predictive value. †Excludes samples from patients with a microbiologic diagnosis of MTBC made at the Mycobacterium Reference Unit in the last 18 or subsequent 3 months, and patients receiving antituberculous treatment currently or within the last 3 months. ‡Includes biopsy specimens from liver (n = 13), kidney (n = 2), skin (n = 15), lung (n = 20), pleura (n = 13), and miscellaneous sites (n = 45). §Includes ascites (n = 56), pericardial aspirates (n = 29), aspirates from miscellaneous sites (n = 80), blood (n = 1), bone marrow (n = 22), feces (n = 1), and urine (n = 15).

**Table 2:** Results of LiPA in detecting rifampin resistance in specimens from which MTBC was correctly identified and cultured*

Sample and AFB smear result	No. positive/no. tested (%)					
	Concordance	Sensitivity	Specificity	PPV	NPV	Mean days saved
All primary LiPA	775/782 (99.1)	38/40 (95.0)	737/740 (99.6)	38/41 (92.7)	738/740 (99.7)	30.7
Positive	743/747 (99.5)	35/36 (97.2)	708/710 (99.7)	35/37 (94.6)	709/710 (99.9)	30.4
Negative	32/35 (91.4)	3/4 (75.0)	29/30 (96.7)	3/4 (75.0)	29/30 (96.7)	37.2
Not done	–	–	–	–	–	–
Respiratory	669/672 (99.6)	32/33 (97.0)	637/639 (99.7)	32/34 (94.1)	637/638 (99.8)	30.4
Positive	654/657 (99.5)	31/32 (96.9)	623/625 (99.7)	31/33 (93.9)	623/624 (99.8)	30.2
Negative	15/15 (100.0)	1/1 (100.0)	14/14 (100.0)	1/1 (100.0)	14/14 (100.0)	39.1
Not done	–	–	–	–	–	–
Nonrespiratory	106/110 (96.4)	6/7 (85.7)	100/101 (99.0)	6/7 (85.7)	101/102 (99.0)	32.5
Positive	89/90 (98.9)	4/4 (100.0)	85/85 (100.0)	4/4 (100.0)	86/86 (100.0)	32.0
Negative	17/20 (85.0)	2/3 (66.7)	15/16 (93.8)	2/3 (66.7)	15/16 (93.8)	35.5
Not done	–	–	–	–	–	–
Clinical isolate	229/235 (97.4)	21/23 (91.3)	208/211 (98.6)	21/24 (87.5)	208/210 (99.0)	16.3

*LiPA, line probe assay; MTBC, *Mycobacterium tuberculosis* complex; AFB, acid-fast bacilli; PPV, positive predictive value; NPV, negative predictive value.

**Table 3:** Final culture identification results for all specimens*

Result	Primary specimens, no. (%)	Isolates, no. (%)	Total, no. (%)
MTBC			
Rifampin sensitive†	892 (94.9)	214 (90.9)	1,106 (93.9)
Rifampin resistant only†	9 (1.0)	3 (1.3)	12 (1.0)
MDRTB†	37 (3.9)	20 (8.4)	57 (4.8)
Susceptibilities not determined†	2 (0.2)	1 (0.4)	3 (0.3)
Total MTBC	940 (47.1)	238 (82.1)	1,178 (51.5)
NTM	181 (9.1)	42 (14.5)	223 (9.8)
Contaminated	22 (1.1)	8 (2.8)	30‡ (1.3)
Culture not done	5 (0.3)	1 (0.3)	6‡ (0.3)
No mycobacterial spp.	849 (42.5)	1 (0.3)	850 (37.2)
Total	1,997	290	2,287

*MTBC, *Mycobacterium tuberculosis* complex; MDRTB, multidrug-resistant tuberculosis; NTM, nontuberculous mycobacteria. †Percentages of total MTBC cultures. ‡These 36 (1.6%) cases without definitive culture results were excluded from analyses of assay performance.

**Table 4:** Mean time in days to culture MTBC from all primary specimens (including those from patients receiving treatment), stratified according to smear microscopy result*

AFB stain result	PCR result			
	Positive (n)	Negative (n)	Equivocal (n)	Total (n)
Positive	18.0 (747)	23.3 (51)	22.0 (9)	18.4 (807)
Negative	25.4 (35)	31.3 (84)	30.0 (6)	29.6 (125)
Not done	0 (0)	22.0 (1)	21.0 (1)	21.5 (2)
Total	18.4 (782)	28.2 (136)	24.9 (16)	19.9 (934)

*MTBC, *Mycobacterium tuberculosis* complex; PCR, polymerase chain reaction; AFB, acid-fast bacilli; n, no. of samples.

### LiPA

Results of LiPA analysis for MTBC were positive in 1,153 (50.4%), negative in 1,085 (47.4%), equivocal in 27 (1.2%), and inhibited in 22 (1.0%) primary specimens. Of the 1,153 PCR-positive samples, 1,085 (94.1%) were reported as rifampin susceptible by LiPA, and 68 (5.9%) were reported as rifampin resistant. Of the 27 PCR-equivocal samples, 16 grew MTBC (6 AFB negative, 1 AFB unknown, 9 AFB positive), 3 grew *M*. *avium* complex, and 8 yielded no mycobacterial growth. Tables 1 and 2, respectively, show the results of LiPA in detecting MTBC from primary specimens and rifampin resistance from specimens that grew MTBC. Data on antituberculous treatment were incomplete, but when reported, 195 (9.8%) samples were from patients receiving treatment currently or within the last 3 months. A total of 309 (15.5%) had a history of antituberculous treatment (Tables 5 and 6).

**Table 5:** Results of LiPA in detecting MTBC in clinical specimens in which MTBC was correctly identified and cultured, stratified by history of antituberculous treatment*

Treatment history/AFB smear result	No. positive/no. tested (%)				
	Concordance	Sensitivity	Specificity	PPV	NPV
Current or within 3 mo	86/182 (47.3)	48/67 (71.6)	38/115 (33.0)	48/125 (38.4)	38/57 (66.7)
Positive	61/132 (46.2)	46/55 (83.6)	15/77 (19.5)	46/108 (42.6)	15/24 (62.5)
Negative/not done	25/50 (50.0)	2/12 (16.7)	23/38 (60.5)	2/17 (11.8)	23/33 (69.7)
Adjusted values†	86/139 (61.9)	48/67 (71.6)	38/72 (52.8)	48/82 (58.5)	38/57 (66.7)
Positive	61/99 (61.6)	46/55 (83.6)	15/44 (34.1)	46/75 (61.3)	15/24 (62.5)
Negative/not done	25/40 (62.5)	2/12 (16.7)	23/28 (82.1)	2/7 (28.6)	23/33 (69.7)
>3 mo ago	85/106 (80.2)	42/53 (79.2)	43/53 (81.1)	42/52 (80.8)	43/54 (79.6)
Positive	54/65 (83.1)	40/45 (88.9)	14/20 (70.0)	40/46 (87.0)	14/19 (73.7)
Negative/not done	31/41 (75.6)	2/8 (25.0)	29/33 (87.9)	2/6 (33.3)	29/35 (82.9)
Adjusted values†	85/102 (83.3)	42/53 (79.2)	43/49 (87.8)	42/48 (87.5)	43/54 (79.6)
Positive	54/63 (85.7)	40/45 (88.9)	14/18 (90.9)	40/44 (90.9)	14/19 (73.7)
Negative/not done	31/39 (79.5)	2/8 (25.0)	29/31 (93.5)	2/4 (50.0)	29/35 (82.5)
No stated treatment	1,497/1,634 (91.6)	692/798 (86.7)	805/836 (96.3)	692/723 (95.7)	805/911 (88.4)
Positive	845/902 (93.7)	661/698 (94.7)	184/204 (90.2)	661/681 (97.1)	184/221 (83.3)
Negative/not done	652/732 (89.1)	31/100 (31.0)	621/632 (98.3)	21/42 (73.8)	621/690 (90.0)
Adjusted values†	1,497/1,634 (91.6)	692/798 (86.7)	805/836 (96.3)	692/723 (95.7)	805/911 (88.4)
Positive	845/892 (94.7)	661/698 (94.7)	184/194 (94.8)	661/671 (98.5)	184/221 (83.3)
Negative/not done	652/728 (89.6)	31/100 (31.0)	621/628 (98.9)	31/38 (81.6)	621/690 (90.0)

*LiPA, line probe assay; MTBC, *Mycobacterium tuberculosis* complex; AFB, acid-fast bacilli; PPV, positive predictive value; NPV, negative predictive value. †Excludes samples from patients with a microbiologic diagnosis of MTBC made at the Mycobacterium Reference Unit in the last 18 or subsequent 3 months.

**Table 6:** Results of LiPA in detecting rifampin resistance in clinical specimens in which MTBC was correctly identified and cultured, stratified by history of antituberculous treatment*

Treatment history	No. positive/no. tested (%)				
	Concordance	Sensitivity	Specificity	PPV	NPV
Current or within 3 mo	46/48 (95.8)	8/10 (80.0)	38/38 (100)	8/8 (100)	38/40 (95.0)
>3 mo ago	41/42 (97.6)	7/7 (100)	34/35 (97.1)	7/8 (87.5)	34/34 (100)
None stated	689/691 (99.7)	23/23 (100)	666/668 (99.7)	23/25 (92.0)	666/666 (100)

*LiPA, line probe assay; MTBC, *Mycobacterium tuberculosis* complex; AFB, acid-fast bacilli; PPV, positive predictive value; NPV, negative predictive value.

### Discrepant Results

There were 136 false-negative MTBC results, i.e., samples negative by LiPA that subsequently yielded MTBC on culture. There were 118 apparently false-positive MTBC results by LiPA, which were PCR positive but did not grow MTBC, although 88 were AFB positive. A total of 83 false-positive samples were considered to have correct molecular results because they were from patients with a microbiologic diagnosis of MTBC made at MRU from another sample (n = 61) or from patients who were receiving antituberculous treatment currently or had received it within the last 3 months (n = 22). These 83 samples were excluded from statistical analysis to give adjusted values for specificity and PPV (Table 1). Ten specimens were from 6 patients with discrepant results for rifampin susceptibility (Table 7). Eight specimens had wild-type *rpoB*, and 2 had mutations not associated with rifampin resistance.

**Table 7:** Sequence analysis of 10 discrepant rifampin-susceptibility results*

Sample no.	Rifampin susceptibility		
	LiPA result	Phenotypic result	Conclusion after sequencing
1–3	Sensitive	Resistant	From the same patient; wild-type *rpoB* test region
4	Sensitive	Resistant	*Mycobacterium bovis*; wild-type *rpoB* test region
5–7	Resistant (DS4)	Sensitive	From the same patient; synonymous substitution (R528R) not associated with rifampin resistance
8	Resistant (DS1)	Sensitive	2 genotypes present: wild-type (predominant) and mutant (L511P)
9	Resistant (R5)	Sensitive	S531L mutation; wild-type *rpoB* on retesting, thus likely laboratory error
10	Resistant (DS2)	Sensitive	D516A mutation; no high-level resistance when seen alone

*LiPA, line probe assay.

## Discussion

We assessed LiPA on the largest reported sample of 1,997 clinical specimens in a nontrial, routine context that would be meaningful to clinicians, especially those submitting samples other than AFB-positive respiratory specimens. The overall unadjusted concordance, sensitivity, specificity, PPV, and NPV were 86.7%, 85.2%, 88.2%, 86.9%, and 86.7%, respectively, for detecting MTBC in primary samples and 98.9%, 98.7%, 100%, 100%, and 93.3%, respectively, for isolates. Previous studies that tested mainly respiratory samples and isolates reported concordance rates with culture from 78.3% to 100% and were usually controlled studies (*8**–**16*).

When PCR was compared with culture for detecting MTBC, some false-positive results may, in fact, have been true-positive results. Of 118 samples classified as false positive, 83 were believed to be true positive on the basis of our planned protocol. These consisted of 61 samples from patients with a microbiologic diagnosis of MTBC at our laboratory in the last 18 or subsequent 3 months and an additional 22 samples from patients who were receiving antituberculous treatment or who had received it within the last 3 months. Patients who were receiving treatment currently or within the last 3 months were significantly less likely to have MTBC; of 195 samples from such patients, 70 (35.9%) had MTBC compared to 871 (49.3%) of 1,766 samples from patients with no reported treatment within the last 3 months (χ^2^ = 12.7, p<0.001). Furthermore, a significantly higher proportion of rifampin-resistant MTBC was isolated from patients receiving treatment (12/70, 17.1%) compared with patients not reported to be receiving treatment (34/866, 3.9%, χ^2^ = 24.2, p<0.001). In these 83 false-positive samples believed to represent true positive results, PCR detected nucleic acid from nonviable organisms (due to treatment) or viable organisms in insufficient numbers for successful culture. If these 83 samples are excluded from overall analysis, specificity improves for all primary specimens, AFB-positive specimens, and AFB-negative specimens from 88.2%, 70.8%, and 95.7%, respectively, to adjusted values of 96.2%, 91.8% and 97.7% (Table 1). PPV improves from 86.9%, 89.5% and 54.7%, respectively, to 95.7%, 97.5% and 70.0%. Other false-positive samples could probably be excluded; we only chose to exclude those with microbiologic diagnoses of MTBC at our laboratory because we had no data on microbiologic, histologic, or clinical diagnoses made by the other hospitals that submitted these samples. Furthermore, since relevant data were often not provided, many more patients likely were receiving antituberculous therapy that we were unaware of because treatment failure is a common reason for specimens being submitted for testing.

PCR-equivocal results were excluded from the primary analysis. However, a PCR-equivocal result may represent a lack of sensitivity. If PCR-equivocal results are considered PCR negative, the adjusted values for detecting MTBC in primary specimens were only marginally altered to 90.6%, 84.0%, 96.2%, 95.7%, and 85.7%, respectively, for concordance, sensitivity, specificity, PPV, and NPV.

A recent review of LiPA results reported that although little data on clinical specimens were available, sensitivity appeared lower than that of isolates (*10*). Our study confirmed this finding, with sensitivities of 85.2% for all clinical specimens and 98.7% for isolates. As with other PCR-based tests (*21**–**23*), sensitivities of LiPA for AFB-negative (29.4%) and nonrespiratory samples (61.1%) were low. Sensitivity was also reduced to 71.6% in patients receiving treatment at the time or within 3 months of the time the sample was obtained. Marttila et al. tested 75 clinical specimens with LiPA, including 66 from nonrespiratory sites, and reported a sensitivity of 58.8% compared with final clinical and pathologic diagnoses, whereas cultures showed a sensitivity of 35.3% (*15*). Several factors may explain the lower sensitivity of PCR-based methods in these samples. The mycobacterial load is lower, as demonstrated by the significantly shorter time taken to culture MTBC for AFB-positive samples than for AFB-negative samples (18.5 days vs. 29.5 days, *z* = 8.0, p<0.001), and respiratory samples than nonrespiratory samples (18.7 days vs. 25.0 days, *z* = 5.6, p<0.001). However, more respiratory samples were AFB positive (94.3% vs. 55.0%). Irregular clumping may take place within paucibacillary specimens, and small, suboptimal sample volumes often lead to sampling errors. Nonrespiratory specimens, especially pleural fluid, bone marrow, pus, and tissue biopsy specimens, may contain inhibitors of amplification (*22**,**23*). Inhibition rates in this study were 1.0% overall, with above-average rates in blood and feces (both 2/3, [66.7%]), pleural fluid (2/110 [1.8%]), bone marrow (1/23 [4.3%]), and pus/tissue (8/400 [2.0%]).

The nonrespiratory specimen types with the highest sensitivity rates were vertebral aspirates/biopsy specimens (n = 30, sensitivity 83.3%), gastric aspirates (n = 18, sensitivity 80.0%), and lymph node aspirates/biopsy specimens (n = 144, sensitivity 72.5%). For pleural fluid, one of the most commonly submitted samples (n = 107), LiPA had one of the lowest sensitivity rates (21.7%) for detecting MTBC. The difficulties in detecting MTBC in pleural fluid are well recognized, with previous reported sensitivities of 20% (*24*) and 50% (*23*) with the Gen-Probe amplified *M*. *tuberculosis* direct test.

For detecting rifampin resistance in PCR-positive specimens yielding MTBC on culture, LiPA had concordance, sensitivity, specificity, PPV, and NPV values of 99.1%, 95.0%, 99.6%, 92.7%, and 99.7%, respectively. These results are consistent with previous studies that reported concordance rates of 90.2% to 100% (*8**–**18*). In this study, of the 69 rifampin-resistant MTBC strains cultured, 5 were PCR negative for MTBC. Of the remaining 64 that were PCR positive, 59 (93.7%) had detectable *rpoB* mutations and were reported as resistant. At least 90% of rifampin-resistant strains have mutations within the target *rpoB* region, although this proportion may vary in different populations (*7*).

Detection of rifampin resistance by LiPA may be used as an early predictor of MDRTB before phenotypic susceptibilities are available, but this clearly depends on the prevalence of rifampin monoresistance in the study population. The diagnosis of rifampin monoresistance is also critical because this automatically invalidates the use of short-course chemotherapy (*25*). Of the 59 correctly identified rifampin-resistant MTBC isolates, 11 were rifampin monoresistant. The overall prevalence was 1.0% in this study, which was higher than the 0.3% reported in a national UK survey (*6*). This result reflects a common underlying reason for specimen referral for Fastrack analysis, i.e., failure of response to treatment.

For primary samples in which LiPA detected MTBC, diagnosis of tuberculosis was made an average of 15.2 days earlier than with automated liquid culture (14.8 days for AFB-positive specimens and 22.1 days for AFB-negative specimens). More days were saved with nonrespiratory samples (18.3 days) than with respiratory samples (14.7 days), although these samples had the lowest probability of detection. LiPA accurately determined rifampin susceptibility earlier than solid culture-based techniques by a mean of 30.7 days for all primary specimens. This compares favorably with a study that found that LiPA saved a median of 24 days compared with susceptibility testing with the BACTEC liquid culture system (Becton Dickinson, Sparks, MD, USA) and 54 days with solid media (*11*).

In summary, LiPA may be used with clinical samples for diagnosis of MTBC and rifampin resistance, saving, when positive results are obtained, an average of 15.2 days and 30.7 days, respectively, compared with conventional techniques. However, some limitations of LiPA are evident. As with other PCR-based assays, sensitivity is reduced in AFB-negative and nonrespiratory samples, such as paucibacillary forms of the disease, in which rapid diagnosis would be most helpful. Although the assay is a potential diagnostic route for patients receiving therapy, sensitivity is also reduced in these circumstances. The lower sensitivity rates for certain samples and the possibility of a PCR-equivocal or PCR-inhibited result also mean that conventional culture and sensitivity testing should still be used at the same time. Alternatives to LiPA may be useful, e.g., we used an IS*6110*-based PCR for diagnosis of tuberculous meningitis. Similarly, rifampin-resistance mutations can be detected by DNA sequencing (we now sequence all PCR products identified as MTBC with any form of rifampin probe mutations) or with noncommercial macroarrays (*26**,**27*). Thus, molecular results, as with any laboratory test, should be reviewed in the context of all clinical, microbiologic, and histologic results.
